# How does the recurrence-related morphology characteristics of the Pcom aneurysms correlated with hemodynamics?

**DOI:** 10.3389/fneur.2023.1236757

**Published:** 2023-10-05

**Authors:** Xiaolong Hu, Peng Deng, Mian Ma, Xiaoyu Tang, Jinghong Qian, Gang Wu, Yuhui Gong, Liping Gao, Rong Zou, Xiaochang Leng, Jianping Xiang, Jiandong Wu, Zhiliang Ding

**Affiliations:** ^1^Department of Neurosurgery, The Affiliated Suzhou Hospital of Nanjing Medical University, Suzhou, Jiangsu, China; ^2^ArteryFlow Technology Co., Ltd., Hangzhou, China

**Keywords:** aneurysm, stent assisted coil embolization, CFD – computational fluid dynamics, recurrence, Pcom aneurysm, hemodynamics

## Abstract

**Introduction:**

Posterior communicating artery (Pcom) aneurysm has unique morphological characteristics and a high recurrence risk after coil embolization. This study aimed to evaluate the relationship between the recurrence-related morphology characteristics and hemodynamics.

**Method:**

A total of 20 patients with 22 Pcom aneurysms from 2019 to 2022 were retrospectively enrolled. The recurrence-related morphology parameters were measured. The hemodynamic parameters were simulated based on finite element analysis and computational fluid dynamics. The hemodynamic differences before and after treatment caused by different morphological features and the correlation between these parameters were analyzed.

**Result:**

Significant greater postoperative inflow rate at the neck (Q_inflow_), relative Q_inflow_, inflow concentration index (ICI), and residual flow volume (RFV) were reported in the aneurysms with wide neck (>4 mm). Significant greater postoperative RFV were reported in the aneurysms with large size (>7 mm). Significant greater postoperative Q_inflow_, relative Q_inflow_, and ICI were reported in the aneurysms located on the larteral side of the curve. The bending angle of the internal carotid artery at the initiation of Pcom (α_ICA@PCOM_) and neck diameter had moderate positive correlations with Q_inflow_, relative Q_inflow_, ICI, and RFV.

**Conclusion:**

The morphological factors, including aneurysm size, neck diameter, and α_ICA@PCOM_, are correlated with the recurrence-inducing hemodynamic characteristics even after fully packing. This provides a theoretical basis for evaluating the risk of aneurysm recurrence and a reference for selecting a surgical plan.

## Introduction

Endovascular therapy (EVT) is a standard treatment for intracranial aneurysms. This method has the advantages of small surgical trauma, fast postoperative recovery, and slight patient pain. Still, the recurrence rate of an aneurysm after surgery is higher than that of surgical clipping (1). Some studies indicated that hemodynamic parameters are essential in the recurrence of treated aneurysms. In general, the recurred aneurysms have more blood flow flowing into the aneurysm through the neck after surgery, which is reflected in larger inflow area (2), inflow concentration index (ICI) (3, 4), residual flow volume (RFV) (2, 5), the average blood flow velocity in the neck region (6–8), and wall shear stress (WSS) (9). Their works promote the explanation of the mechanism of aneurysm recurrence. High-flow blood may prevent the formation of a thrombus, increase the instability of the coil cluster and promote aneurysm growth, and then lead to aneurysm recurrence.

The recurrence rate of the posterior communicating artery (Pcom) aneurysms is particularly higher than that for aneurysms in other sites (1, 10, 11). This may be related to the unique position and structure of Pcom. Some studies showed that morphological factors are associated with the recurrence risk, such as aneurysm volume (12), neck diameter (13), the bending angle of the internal carotid artery (ICA) at the initiation of Pcom (α_ICA@PCOM_), Pcom diameter, and whether the aneurysm belongs to Pcom-incorporated type (the neck mainly distributed in Pcom) are related to the recurrence of Pcom aneurysms (13–16). However, the mechanism by which these morphological features induce recurrence is not clear. This study aims to investigate whether these morphological features induce recurrence in a hemodynamic way.

## Methods

### Patient selection

The present study was conducted at our hospital and involved a retrospective review of patients admitted to the Stroke Center from November 2019 to March 2022. Patients with Pcom aneurysms confirmed by digital subtraction angiography (DSA) with dense embolization were enrolled in this study. Informed consent was obtained from all patients, and the study was approved by the ethics review committee of the hospital. The inclusion and exclusion criteria for this study were as follows:

Inclusion criteria: (1) Patients with Pcom aneurysms, (2) Complete imaging data sufficient to construct a complete vascular model, and (3) Aneurysms treated by coil embolization with or without stent-assisted. Exclusion criteria: (1) Infectious aneurysms, (2) Dissecting aneurysms, (3) Traumatic aneurysms, and (4) Incomplete imaging data.

### Model reconstruction and simulation analysis

The reconstruction of the vascular and aneurysm models relied on 3D-DSA images in DICOM format obtained from patients including pre-operation and post-operation, with subsequent trimming and smoothing of the STL model carried out in Geomagic Wrap 2015 (Research Triangle Park, North Carolina, United States). This process involved removing minor branches from the vascular model and retaining the aneurysm, the parent vessel, and critical branching vessels.

The virtual implantation of the stent and coil adopts the method established in previous studies (17). Enterprise, Neuroform EZ and LVIS stents were modeled using NX 12.0 (Siemens PLM Software, Plano, TX, United States). The stent simulation was divided into three stages: compression, delivery, and deployment, all of which were realized by employing ABAQUS version 6.14 (SIMULIA, Providence, Rhode Island, United States). Coils were generated in MATLAB (MathWorks, Natwick, MA). The embolization of coils was conducted in two steps, pulling the coils into the microcatheter and pushing it into the aneurysm following the order in the actual operation process.

The reconstructed vascular model and the finite element models of the stents and coils were used for hemodynamic simulation. The vascular, stent and coil models were imported into ANSYS ICEM CFD version 16.2 (ANSYS Inc., Canonsburg, PA, United States) for meshing, with a global mesh size of 0.16 mm. The surface mesh size of the Enterprise and Neuroform EZ stents was set to 0.03 mm, the mesh size of the LVIS stent was set to 1/6 of the wire circumference, which was 0.03 mm, and the mesh size of the coils was set to 0.1 mm (18). The CFD simulation was based on the Navier–Stokes equations and was performed using ANSYS CFX version 2019 (ANSYS Inc., Canonsburg, PA, United States). Blood was modeled as an incompressible, laminar, Newtonian fluid with a density of 1,056 kg/m^3^ and a viscosity of 0.0035 kg/m·s. The vascular wall was set as a rigid and no-slip boundary condition. The inlet flow rate was set to 4.6 mL/s, and the outlet flow rate was calculated according to the Murray flow rate distribution law (19).

The calculated hemodynamic parameters including the inflow rate at the neck (Q_inflow_), the relative inflow rate at the neck (relative Q_inflow_), ICI, the WSS of the aneurysm and the parent vessel (WSSa and WSSp), the average velocity of the aneurysm sac (Va), RFV in the aneurysm sac. The relative Q_inflow_ is defined as the ratio of the inflow rate at the neck to that of the parent vessel. The RFV was measured based on four threshold values 0.05 m/s, 0.10 m/s, 0.15 m/s, and 0.2 m/s, respectively.

### Morphological parameters measurement

**Figure 1:**
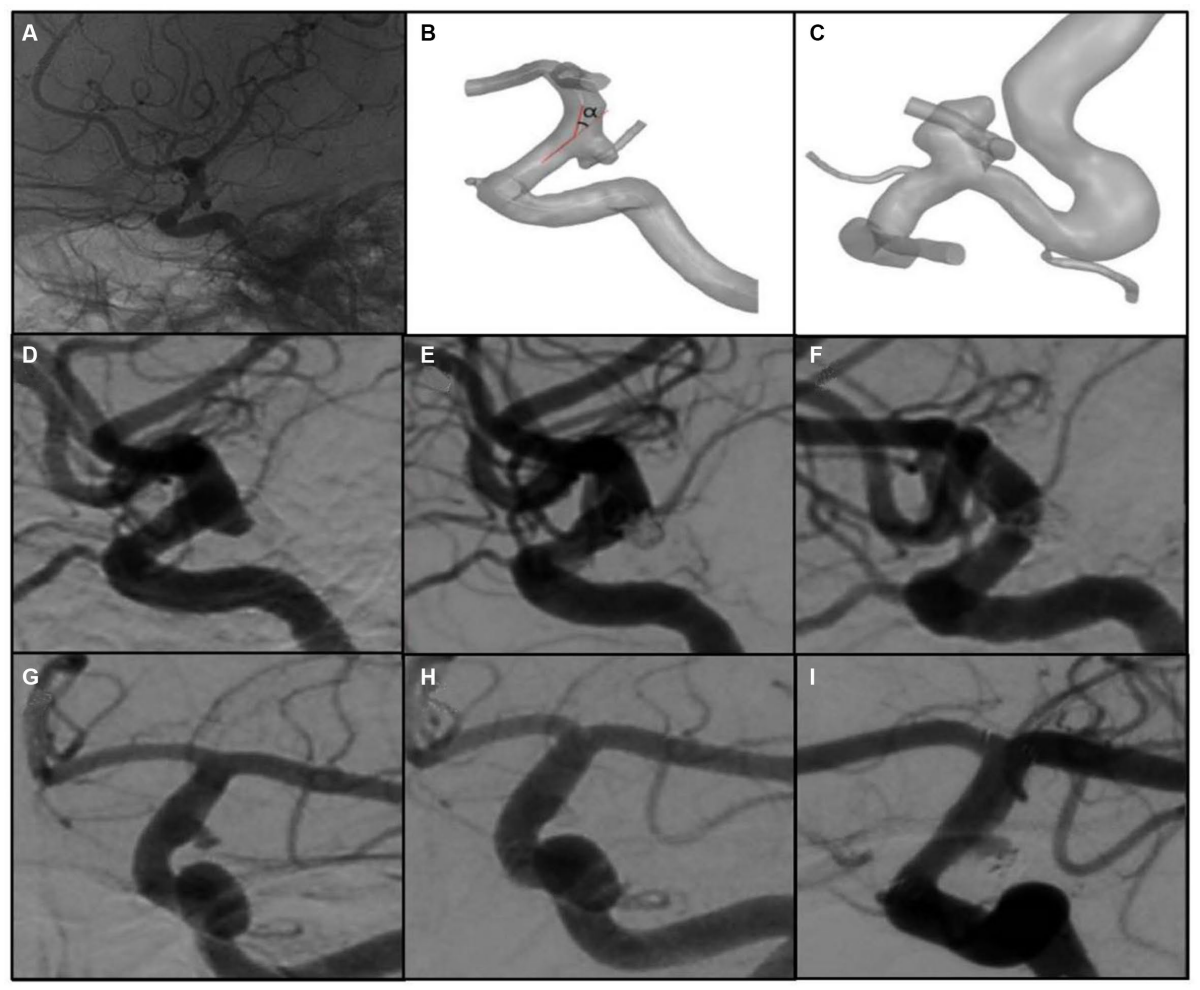
Measurement of α_ICA@PCOM_, location of aneurysm and angiography images of aneurysms. **(A)** The 2D-DSA image of the aneurysm. **(B)** The bending angle of ICA at the aneurysm (α_ICA@PCOM_). **(C)** Aneurysm locates on the lateral side of the curve. **(D–F)** are pre- and post-operative and follow-up images of a non-recurrent case, respectively. **(G–I)** are pre- and post-operative and follow-up images of a recurrent case, respectively.

Aneurysm size was defined as the maximum height. The maximum height, the neck diameter and Pcom diameter were measured in the reconstructed model. Adopting a methodology similar to Rosato’s research (14), the α_ICA@PCOM_ is defined, as shown in Figure 1. The centerline of the internal carotid artery (ICA) is captured based on a 3D model, and the angle between the approximate straight segments of the local centerline of ICA is taken as the bending angle of ICA. The apex of the angle is located at the projection of the centroid of the aneurysm neck on the centerline. According to the different positions of the aneurysm in the direction of vascular curve, the aneurysms were divided into those located on the lateral side of the curve and those located in other positions (Figure 1C shows the aneurysm located on the lateral side).

### Statistical analysis

The data analysis between morphological and hemodynamic parameters was performed using SPSS 25.0 (IBM Corp, Chicago, IL, United States). Continuous variables were presented with mean and standard deviation and categorical variables were presented with number and percentage. All the aneurysms were grouped according to maximum height (whether the maximum height > 7 mm) (20), neck diameter (whether the neck diameter > 4 mm) (21), whether the aneurysm belongs to Pcom-incorporated type or locates on the lateral side, respectively. Univariate analysis to evaluate the relevant factors for hemodynamical parameters was performed using Student’s *t*-test. In addition, Pearson analysis used to depict the correlations between morphological parameters and hemodynamics. A *p* value <0.05 was considered statistically significant, and a *r* value >0.5 was considered that there is a correlation between the two variables.

## Results

### Clinical results

A total of 20 patients with 22 aneurysms were enrolled in this study, including 10 (45.5%) ruptured aneurysms and 12 (54.5%) unruptured aneurysms, all of whom underwent coil embolization with or without stent-assisted. Among them, six aneurysms underwent simple coil embolization, and 16 underwent stent-assisted coiling. All cases had a coil packing density ranging from 25 to 30% (coil packing density was defined as the ratio of coils’ volume to the aneurysm’s volume). Post-operation angiography showed complete occlusion in 19 (86.4%) of the 22 aneurysms, and residual neck in 3 (13.6%) aneurysms. With the follow-up to patients, 3 (13.6%) recurrent cases were found. One was treated with stent-assisted coil embolization. Two had residual neck in post-operation angiography. All these aneurysms were Pcom-incorporated and two were on the lateral side of the vessel. The angiography images of one recurrent case at pre-operation, post-operation and follow-up status were shown in Figures 1G–I. The basic information of all 20 patients with 22 aneurysms is shown in Table 1.

**Table 1:** Characteristics information for the patients.

Characteristics	Value
Clinical features	Patients (*n* = 20)
Male	6 (30%)
Mean age	65.3
Hypertension	13 (65%)
Diabetes	4 (20%)
Smoking	2 (10%)
Drinking	2 (10%)
Aneurysm information	Aneurysms (*n* = 22)
Rupture	10 (45%)
Stent-assisted coiling	16 (73%)
Pcom-incorporated	6 (27%)
Lateral	11 (50%)
Pcom diameter (mean ± SD, mm)	1.21 ± 0.62
Neck diameter (mean ± SD, mm)	4.01 ± 1.28
Maximum height (mean ± SD, mm)	5.20 ± 2.72
α_ICA@PCOM_ (mean ± SD, °)	56.68 ± 16.48

### Morphological parameters and hemodynamic results

Statistically significant results of univariate analysis are shown in Table 2. All hemodynamic parameters were reduced after coiling. However, the aneurysms with large size, wide neck or on the lateral side had larger blood flowing into aneurysm (Q_inflow_, relative Q_inflow_, and ICI) and RFV, whether pre- or post-operative. Meanwhile, these aneurysms had a smaller reduction of Q_inflow_, relative Q_inflow_ and ICI after coiling. The reduction rates of RFV in all groups were close. Correlation analysis of α_ICA@PCOM_, maximum height and neck diameter showed in Table 3. α_ICA@PCOM_ was statistically correlated (*r* > 0.5, *p* < 0.05) with both pre- and post-operation RFV, and with post-operation Q_inflow_ and relative Q_inflow_. Maximum height was statistically correlated with pre-operation A_inflow_, relative Q_inflow_, ICI, and RFV, and with post-operation A_inflow_ and relative Q_inflow_. Neck diameter was statistically correlated with both pre- and post-operation A_inflow_, Q_inflow_, relative Q_inflow_, ICI, and RFV.

**Table 2:** Univariate analysis of factors related to hemodynamical parameters.

			Q_inflow_ (ml/s)	Relative Q_inflow_	ICI	RFV (mm^3^)			
						v > 0.05 m/s	v > 0.1 m/s	v > 0.15 m/s	v > 0.2 m/s
Maximum Height	<7 mm	Pre	1.29	0.3	0.66	31.83*	30.50*	28.54*	25.42*
		Post	0.62	0.14	0.4	8.31*	5.42*	3.86*	2.81*
		Change	51%	53%	39%	74%	82%	86%	89%
	>7 mm	Pre	1.83	0.43	0.99	141.7*	124.7*	105.4*	79.23*
		Post	1.31	0.3	0.65	31.54*	20.35*	14.43*	11.08*
		Change	28%	30%	34%	78%	84%	86%	86%
Neck Diameter	<4 mm	Pre	0.98*	0.22*	0.44*	33.65	28.08*	22.95*	16.99*
		Post	0.44*	0.10*	0.25*	6.07*	3.76*	2.56*	1.77*
		Change	55%	55%	43%	82%	87%	89%	90%
	>4 mm	Pre	1.93*	0.46*	1.09*	84.58	80.52*	73.68*	62.44*
		Post	1.18*	0.28*	0.70*	22.62*	14.88*	10.72*	8.19*
		Change	39%	39%	36%	73%	82%	86%	87%
Lateral	Yes	Pre	1.69	0.4	0.95	84.81*	76.01*	66.02*	52.75
		Post	1.11*	0.26*	0.68*	20.27	13.3	9.26	6.87
		Change	34%	35%	28%	76%	83%	86%	87%
	No	Pre	1.13	0.26	0.52	28.8*	27.83*	26.0*	22.55
		Post	0.44*	0.1*	0.23*	6.92	4.33	3.28	2.51
		Change	61%	62%	56%	76%	84%	87%	89%

Lateral: the aneurysm locates on the lateral side of the curve; Q_inflow_: the inflow rate at the neck; Relative Q_inflow_: relative inflow rate at the neck; ICI: inflow concentration index; RFV: volume of blood flow in the aneurysm where velocity is larger than 0.05 m/s, 0.10 m/s, 0.15 m/s, and 0.2 m/s, respectively. Change represents the reduction rate of hemodynamical parameters after operation, defined as pre-operation – post-operation/pre-operation.

Values with * are those with *p*-values smaller than 0.05.

**Table 3:** Correlation analysis between morphological and hemodynamic parameters.

Variables		α_ICA@PCOM_		Maximum height		Neck diameter	
		*r* value					
		Pre	Post	Pre	Post	Pre	Post
Q_inflow_		0.420	0.531*	0.487*	0.499*	0.704*	0.678*
Relative Q_inflow_		0.401	0.511*	0.502*	0.507*	0.741*	0.707*
RFV	v > 0.05 m/s	0.566*	0.571*	0.875*	0.483*	0.785*	0.625*
	v > 0.1 m/s	0.565*	0.560*	0.843*	0.439*	0.851*	0.588*
	v > 0.15 m/s	0.569*	0.542*	0.803*	0.444*	0.885*	0.616*
	v > 0.2 m/s	0.583*	0.517*	0.717*	0.467*	0.888*	0.664*

Correlation coefficients with * are those with *p*-values smaller than 0.05.

No statistically significant result with hemodynamic characteristics was found for Pcom incorporated type or Pcom diameter. Complete analysis results were shown in Supplementary Material.

### CFD results of illustrative cases

**Figure 2:**
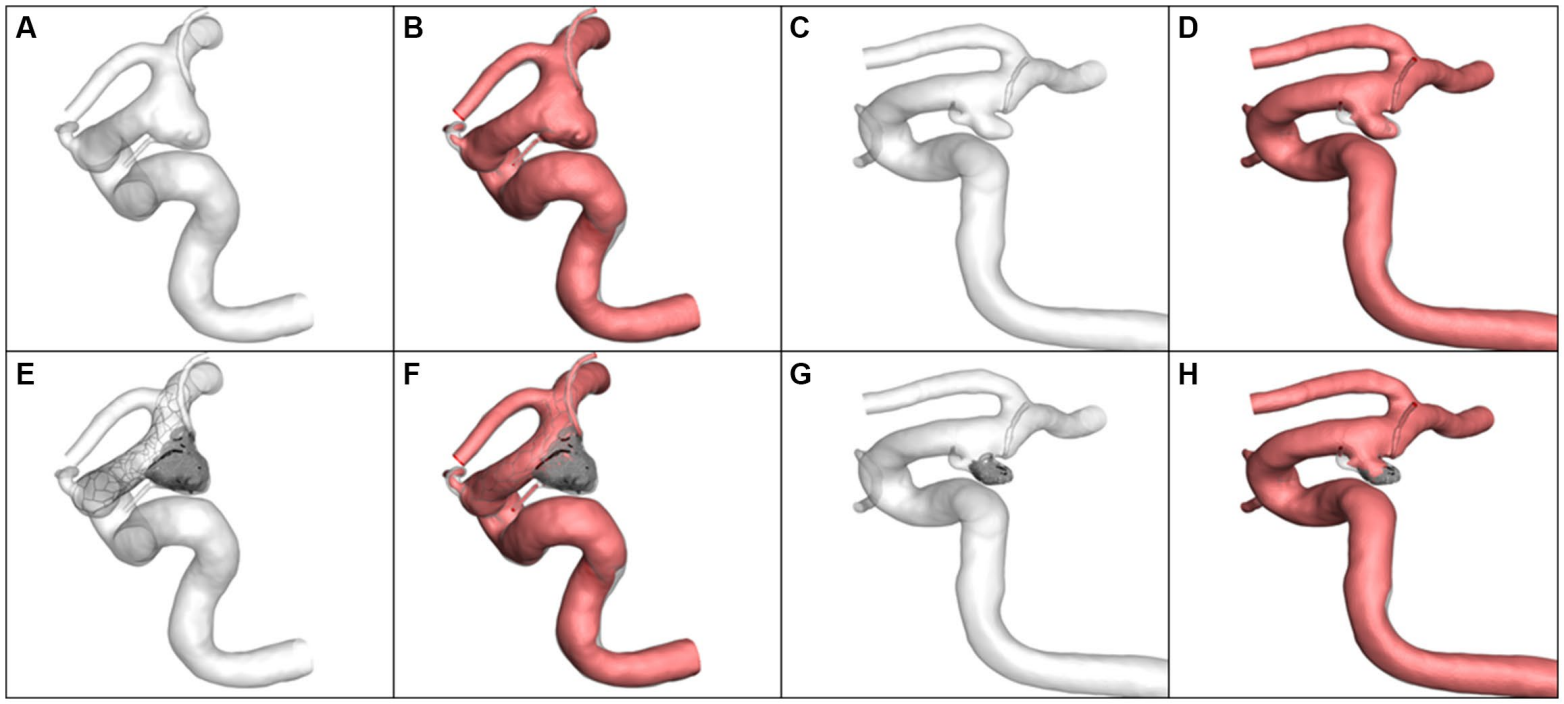
The simulation results and volume of blood flow velocity are larger than 0.1 m/s. **(A,B)** Case 1 pre-operation, **(C,D)** Case 2 pre-operation, **(E,F)** Case 1 post-operation, **(G,H)** Case 2 post-operation.

**Figure 3:**
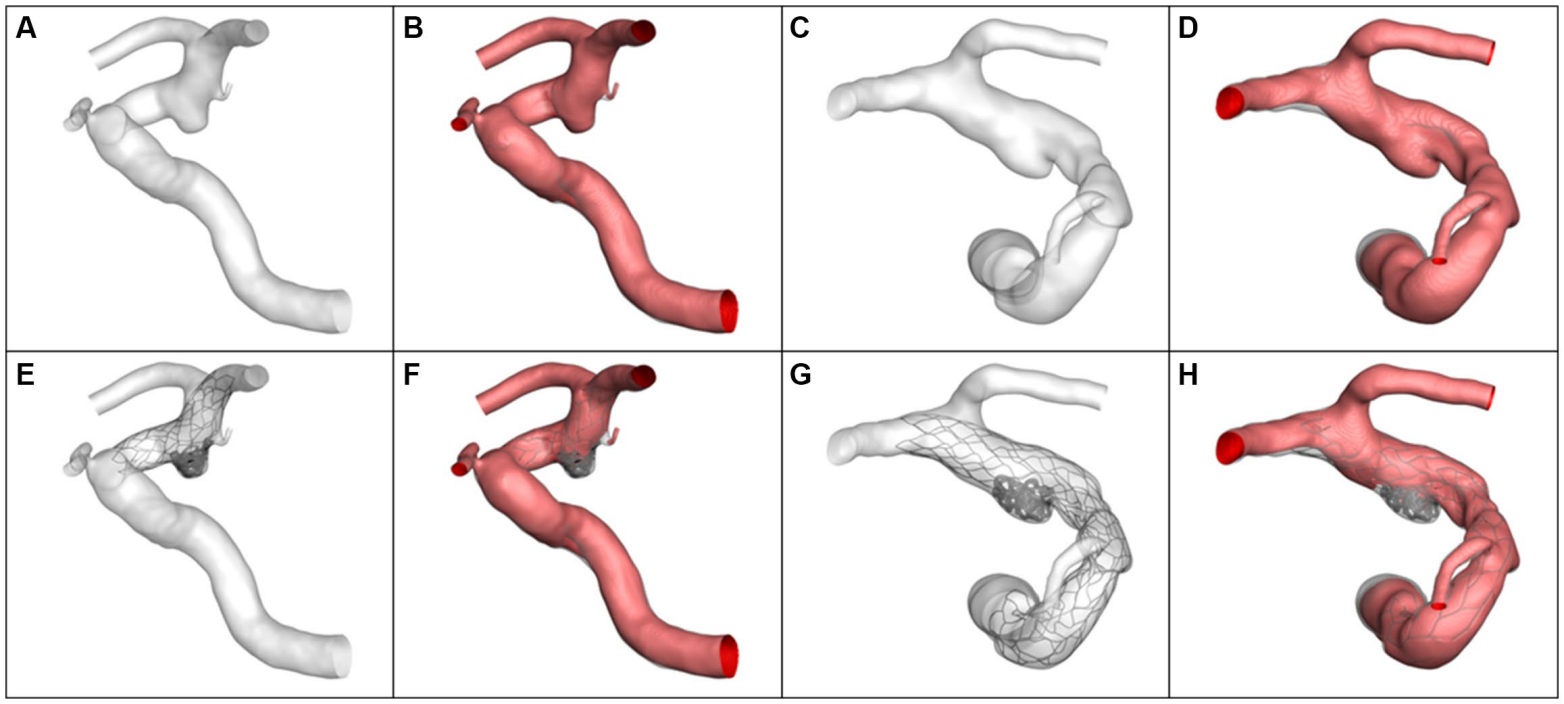
The simulation results and volume of blood flow velocity are larger than 0.1 m/s. **(A,B)** Case 3 pre-operation, **(C,D)** Case 4 pre-operation, **(E,F)** Case 3 post-operation, **(G,H)** Case 4 post-operation.

Four typical cases were identified in which the calculated results were consistent with the statistical analysis. The morphological differences between the aneurysms in Case 1 and Case 2 were reflected in the aneurysm neck diameter and maximum height. Case 1 had a larger neck diameter (5.82 mm vs. 2.89 mm) andmaximum height (8.39 mm vs. 4.60 mm). The hemodynamic calculation results before and after virtual embolization, including Q_inflow_, relative Q_inflow_, ICI, RFV, all showed larger values in Case 1 compared to Case 2, as visualized in Figure 2. Meanwhile, the differences in morphology between Case 3 and Case 4 were manifested in the α_ICA@PCOM_. Case 3 had a larger α_ICA@PCOM_ than Case 4 (41.0° vs. 27.1°). The hemodynamic calculation results for relative Q_inflow_, and RFV were larger for Case 3 compared to Case 4, as shown in Figure 3.

## Discussion

Morphological and hemodynamic parameters of inducing aneurysm recurrence have been reported in numerous studies (12–16, 22–26). This study focuses on investigate whether these morphological features induce recurrence in a hemodynamic mechanism. The study revealed that the morphological factors, including aneurysm size, neck diameter, locating on the lateral side of the curve, and α_ICA@PCOM_, affect hemodynamic parameters pre- and post-operative, such as Q_inflow_, relative Q_inflow_, ICI, and RFV.

The degree of curve of ICA at the initiation of Pcom characterizes the deviation of blood flow direction at this position. By qualitatively determining whether the aneurysm is located on the lateral side of the curve, it can also effectively reflect whether the aneurysm is susceptible to impact during changes in blood flow direction. In this study, as α_ICA@PCOM_ increased, a stronger blood flow jet passed through the aneurysm neck, which makes aneurysms have similar characteristics to bifurcation aneurysms and more prone to initiation, growth and recurrence. Hu et al. (27) have found that the angle between the C6 and C7 segments of the ICA is an independent factor correlated with aneurysm presence. Rosato et al. (14) also provide evidence in favor of the conclusions drawn by Hu et al., highlighting the significant role of wall shear stress in the formation of aneurysms. Lauric et al. (28) suggest that curved blood vessels experience a greater impact on the vessel wall from blood flow at the curve, which may lead to pathological changes favorable for the formation of aneurysms. The initiation of an anterior communicating artery aneurysm may increase with a smaller angle between the A1 and A2 segments of the artery and a more significant difference in angle between the two sides (29). The present study identified that α_ICA@PCOM_ had moderate positive correlations with Q_inflow_, relative Q_inflow_, and RFV. And the aneurysm on the lateral side of the curve had statistically significant larger Q_inflow_, relative Q_inflow_, ICI, and RFV. This may cause the instability of the coils near the neck and the existence of a larger high velocity area near the neck, and increase the possibility of recurrence in the future. Kim et al. (30) reported an outer curve aneurysm with lower vascular tortuosity was more similar to a bifurcation aneurysm and more likely to recur. Leng et al. (31) and Wan et al. (32) both pointed out that better hemodynamic changes can be achieved by using stents to straighten blood vessels. The risk of recurrence can be reduced by weakening the hemodynamic characteristics of bifurcation aneurysms, which was also consistent with our research.

The aneurysm size is widely considered a recurrence risk factor (33, 34). Fukuta et al. (13) suggested that aneurysm neck size is a potential risk factor for the recurrence of Pcom aneurysms. Lee et al. (12) showed a significant correlation between aneurysm volume and recurrence. In our study, the influence of the aneurysm size and the neck diameter on the hemodynamics was analyzed. The aneurysms with neck diameter > 4 mm had statistically significant larger Q_inflow_, relative Q_inflow_, ICI, and RFV, and those with size >7 mm had statistically significant larger RFV. There are more gaps between coils in large aneurysms, and the larger neck also increases the possibility of receiving the impact of high-velocity blood flow and more space between the coils to let blood flow through.

The aneurysm neck is mainly distributed in Pcom, which is also more prone to develop a turbulent flow with more complex hemodynamics near the aneurysm neck, leading to a higher risk of recurrence (16). However, in this study, there was no significant result related to the Pcom-incorporated aneurysm. In recent studies, the association between Pcom aneurysm recurrence and fetal-type Pcom has remained controversial (12, 23). Lee et al. (12) demonstrated that fetal-type Pcom might be an independent risk factor for the recurrence of Pcom aneurysms. Still, Kim et al. suggested that fetal-type Pcom was associated with aneurysm size but not with the risk of rupture or recanalization (23). In general, fetal-type Pcom is larger in diameter with a larger flow rate. Some research indicated that the diameter of Pcom can affect blood supply and thus impact the recurrence of aneurysms (13, 15). In our studies, fetal-Pcom was found in one recurrent case of three recurrent cases, and no hemodynamic parameter was correlated with Pcom diameter.

Huang et al. found that the recurrence rate of an aneurysm was higher when the packing density was lower than 20% (35). However, in our study, although we controlled the coil packing density of all cases ranging from 25 to 30%, hemodynamic parameters still showed a positive correlation with the volume. It is worth noting that for aneurysms with large aneurysm size, wide neck, and on the lateral side of the curve, even if the standard of dense packing based on experience is met, the hemodynamic parameters may be still significantly higher than those of typical aneurysms. This means that the recurrence mechanism of aneurysms with the above characteristics is closely related to hemodynamics. When aneurysms with these characteristics require endovascular treatment, more consideration needs to be given to changing blood flow conditions, such as straighten the parent vessels, fully filling the neck of the aneurysm, and using flow diverters, or adopt clipping to reduce the recurrence rate.

There are several limitations to this study. Firstly, the sample size was relatively small, including only 22 aneurysms from 20 patients. The limited number of recurrent cases limits our subgroup analysis of recurrent cases. Secondly, the lack of patient-specific flow rate information may weak the reliability of the conclusions, especially for the fetal type Pcom. Therefore, a prospective study with a larger sample size and more comprehensive information may be necessary.

## Conclusion

The morphological characteristics of Pcom aneurysms, including aneurysm size, neck diameter, locating on the lateral side of the curve and α_ICA@PCOM_ induce aneurysm recurrence through hemodynamic mechanisms. When treating Pcom aneurysms with large size, wide neck, or on the lateral side of the curve, more consideration needs to be given to changing blood flow conditions. This provides a theoretical basis for evaluating the risk of aneurysm recurrence and a reference for selecting a surgical plan.

## Data availability statement

The original contributions presented in the study are included in the article/Supplementary material, further inquiries can be directed to the corresponding authors.

## Ethics statement

The studies involving humans were approved by Independent Ethic Committee of SuZhou Municipal Hospital. The studies were conducted in accordance with the local legislation and institutional requirements. Written informed consent for participation was not required from the participants or the participants’ legal guardians/next of kin in accordance with the national legislation and institutional requirements.

## Author contributions

XH, ZD, and PD conceived of the presented idea. JQ, GW, YG, and MM perform this study. XH, XT, and JW contributed significantly to analysis and manuscript preparation. LG, RZ, XL, and JX performed the data analysis. XH wrote the manuscript with support from PD and ZD helped supervise the project. JW, ZD, and XH helped performed the analysis with constructive discussions. All authors contributed to the article and approved the submitted version.
