# Supplementary material for: How does the recurrence-related morphology characteristics of the Pcom aneurysms correlated with hemodynamics?
Source: Front Neurol. 2023 Oct 5;14:1236757. doi: 10.3389/fneur.2023.1236757 (PMC10585265; doi:10.3389/fneur.2023.1236757)
Supplement: Supplementary file 1 [file Table_1.DOCX]

Univariate analysis was performed to evaluate the relationships for maximum height, neck diameter and whether an aneurysm belongs to Pcom incorporated type or locates on the lateral side of the curve to hemodynamic parameters, and the results are as shown in Table 1 and Table 2.

Table 1. The mean and reduction rate of hemodynamic parameters compared by maximum height and neck diameter.

|  | | **Maximum height <7mm** | | | **Maximum height >7mm** | | | **Neck diameter <4mm** | | | **Neck diameter >4mm** | | |
| --- | --- | --- | --- | --- | --- | --- | --- | --- | --- | --- | --- | --- | --- |
|  | | **pre** | **post** | **reduction rate** | **pre** | **post** | **reduction rate** | **pre** | **post** | **reduction rate** | **pre** | **post** | **reduction rate** |
| **A_inflow_ (mm^2^)** | | 6.22* | 4.27* | 31.35% | 10.65* | 10.12* | 4.98% | 5.26* | 3.58* | 31.94% | 9.58* | 8.03* | 16.18% |
| **Q_inflow_ (ml/s)** | | 1.29 | 0.62 | 51.94% | 1.83 | 1.31 | 28.42% | 0.98* | 0.44* | 55.10% | 1.93* | 1.18* | 38.86% |
| **Relative Q_inflow_** | | 0.30 | 0.14 | 53.33% | 0.43 | 0.30 | 30.23% | 0.22* | 0.10* | 54.55% | 0.46* | 0.28* | 39.13% |
| **ICI** | | 0.66 | 0.40 | 39.39% | 0.99 | 0.65 | 34.34% | 0.44* | 0.25* | 43.18% | 1.09* | 0.70* | 35.78% |
| **WSSa(Pa)** | | 7.04 | 2.31 | 67.19% | 3.95 | 1.19 | 69.87% | 6.21 | 2.41 | 61.19% | 6.48 | 1.63 | 74.85% |
| **WSSp(Pa)** | | 9.90 | 9.05 | 8.59% | 7.62 | 6.92 | 9.19% | 9.08 | 8.32 | 8.37% | 9.7 | 8.86 | 8.66% |
| **Va(m/s)** | | 0.30 | 0.07 | 76.67% | 0.22 | 0.06 | 72.73% | 0.27 | 0.07 | 74.07% | 0.29 | 0.07 | 75.86% |
| **RFV**  **(mm^3^)** | **v>0.05m/s** | 31.83* | 8.31* | 73.89% | 141.71* | 31.54* | 77.74% | 33.65 | 6.07* | 81.96% | 84.58 | 22.62* | 73.26% |
|  | **v>0.1m/s** | 30.50* | 5.42* | 82.23% | 124.73* | 20.35* | 83.68% | 28.08* | 3.76* | 86.61% | 80.52* | 14.88* | 81.52% |
|  | **v>0.15m/s** | 28.54* | 3.86* | 86.48% | 105.40* | 14.43* | 86.31% | 22.95* | 2.56* | 88.85% | 73.68* | 10.72* | 85.45% |
|  | **v>0.2m/s** | 25.42* | 2.81* | 88.95% | 79.23* | 11.08* | 86.02% | 16.99* | 1.77* | 89.58% | 62.44* | 8.19* | 86.88% |

A_inflow_: the inflow area at the neck; Q_inflow_: the inflow rate at the neck; Relative Q_inflow_: relative inflow rate at the neck; ICI: inflow concentration index; WSSa: the wall shear stress of the aneurysm wall; WSSp: the wall shear stress of the parent artery; Va: the average blood flow velocity in aneurysm; RFV: volume of blood flow in the aneurysm where velocity is larger than 0.05m/s, 0.10m/s, 0.15m/s, 0.2m/s, respectively.

Mean values with * are those with p-values smaller than 0.05.

Table 2. The mean and reduction rate of hemodynamic parameters compared by whether aneurysm belongs to Pcom incorporated type or locates on the lateral side of the curve.

|  | | **Pcom incorporated** | | | **Pcom unincorporated** | | | **Lateral** | | | **Not lateral** | | | |
| --- | --- | --- | --- | --- | --- | --- | --- | --- | --- | --- | --- | --- | --- | --- |
|  |  | **pre** | **post** | **reduction rate** | **pre** | **post** | **reduction rate** | **pre** | **post** | **reduction rate** | **pre** | **post** | **reduction rate** |  |
| **A_inflow_ (mm^2^)** | | 6.36 | 5.89 | 7.39% | 7.55 | 5.49 | 27.28% | 7.6 | 6.11 | 19.61% | 6.85 | 5.09 | 25.69% |  |
| **Q_inflow_ (ml/s)** | | 1.28 | 0.94 | 26.56% | 1.46 | 0.72 | 50.68% | 1.69 | 1.11* | 34.32% | 1.13 | 0.44* | 61.06% |  |
| **Relative Q_inflow_** | | 0.28 | 0.2 | 28.57% | 0.35 | 0.17 | 51.43% | 0.4 | 0.26* | 35.00% | 0.26 | 0.1* | 61.54% |  |
| **ICI** | | 0.54 | 0.41 | 24.07% | 0.81 | 0.47 | 41.98% | 0.95 | 0.68* | 28.42% | 0.52 | 0.23* | 55.77% |  |
| **WSSa (Pa)** | | 5.45 | 2.74 | 49.72% | 6.67 | 1.8 | 73.01% | 6.43 | 1.99 | 69.05% | 6.24 | 2.11 | 66.19% |  |
| **WSSp (Pa)** | | 8.75 | 7.95 | 9.14% | 9.62 | 8.79 | 8.63% | 10.6 | 10.29 | 2.92% | 8.16 | 6.84 | 16.18% |  |
| **Va (m/s)** | | 0.26 | 0.1 | 61.54% | 0.29 | 0.06 | 79.31% | 0.28 | 0.07 | 75.00% | 0.28 | 0.07 | 75.00% |  |
| **RFV**  **(mm^3^)** | **v>0.05m/s** | 41.54 | 18.39 | 55.73% | 62.53 | 11.79 | 81.15% | 84.81* | 20.27 | 76.10% | 28.8* | 6.92 | 75.97% |  |
|  | **v>0.1m/s** | 38.35 | 12.64 | 67.04% | 57.01 | 7.38 | 87.05% | 76.01* | 13.3 | 82.50% | 27.83* | 4.33 | 84.44% |  |
|  | **v>0.15m/s** | 33.15 | 8.72 | 73.70% | 50.83 | 5.35 | 89.47% | 66.02* | 9.26 | 85.97% | 26.0* | 3.28 | 87.38% |  |
|  | **v>0.2m/s** | 27.67 | 6.22 | 77.52% | 41.39 | 4.11 | 90.07% | 52.75 | 6.87 | 86.98% | 22.55 | 2.51 | 88.87% |  |

Pcom: posterior communicating artery; Pcom-incorporated: aneurysms with neck mainly distributed on Pcom; A_inflow_: the inflow area at the neck; Q_inflow_: the inflow rate at the neck; Relative Q_inflow_: relative inflow rate at the neck; ICI: inflow concentration index; WSSa: the wall shear stress of the aneurysm wall; WSSp: the wall shear stress of the parent artery; Va: the average blood flow velocity in aneurysm; RFV: volume of blood flow in the aneurysm where velocity is larger than 0.05m/s, 0.10m/s, 0.15m/s, 0.2m/s, respectively.

Mean values with * are those with p-values smaller than 0.05.

To explore the relationship of the Pcom diameter and hemodynamic parameters and α_ICA@PCOM_ and hemodynamic parameters, correlation analysis was performed and the results are shown in Table 3.

Table 3. Correlation analysis between morphological parameters and hemodynamic parameters.

| **Variables** | | **Pcom diameter** | | **α_ICA@PCOM_** | | **Maximum height** | | **Neck diameter** | |
| --- | --- | --- | --- | --- | --- | --- | --- | --- | --- |
|  | | **r value** | | | | | | | |
|  | | **pre** | **post** | **pre** | **post** | **pre** | **post** | **pre** | **post** |
| **A_inflow_** | | 0.411 | 0.428* | 0.151 | 0.266 | 0.542* | 0.582* | 0.826* | 0.762* |
| **Q_inflow_** | | 0.134 | 0.207 | 0.420 | 0.531* | 0.487* | 0.499* | 0.704* | 0.678* |
| **Relative Q_inflow_** | | 0.151 | 0.193 | 0.401 | 0.511* | 0.502* | 0.507* | 0.741* | 0.707* |
| **ICI** | | 0.089 | 0.097 | 0.463* | 0.466* | 0.506* | 0.410* | 0.766* | 0.664* |
| **WSSa** | | -0.309 | -0.122 | 0.058 | -0.177 | -0.268 | -0.415 | 0.014 | -0.306* |
| **WSSp** | | -0.143 | -0.206 | 0.086 | 0.198 | -0.074 | -0.013 | 0.124 | 0.093 |
| **Va** | | -0.281 | -0.003 | 0.048 | -0.006 | -0.210 | -0.177 | 0.097 | -0.064 |
| **RFV** | **v>0.05m/s** | 0.241 | 0.347 | 0.566* | 0.571* | 0.875* | 0.483* | 0.785* | 0.625* |
|  | **v>0.1m/s** | 0.252 | 0.337 | 0.565* | 0.560* | 0.843* | 0.439* | 0.851* | 0.588* |
|  | **v>0.15m/s** | 0.222 | 0.331 | 0.569* | 0.542* | 0.803* | 0.444* | 0.885* | 0.616* |
|  | **v>0.2m/s** | 0.149 | 0.330 | 0.583* | 0.517* | 0.717* | 0.467* | 0.888* | 0.664* |

Pcom: posterior communicating artery; A_inflow_: the inflow area at the neck; Q_inflow_: the inflow rate at the neck; Relative Q_inflow_: relative inflow rate at the neck; ICI: inflow concentration index; WSSa: the wall shear stress of the aneurysm wall; WSSp: the wall shear stress of the parent artery; Va: the average blood flow velocity in aneurysm; RFV: volume of blood flow in the aneurysm where velocity is larger than 0.05m/s, 0.10m/s, 0.15m/s, 0.2m/s, respectively.

Correlation coefficients with * are those with p-values smaller than 0.05.
